# CHIP-mediated ubiquitination of Galectin-1 predicts colorectal cancer prognosis

**DOI:** 10.7150/ijbs.41125

**Published:** 2020-01-14

**Authors:** Weimin Wang, Zhen Zhou, Liangliang Xiang, Mengying Lv, Tengyang Ni, Jianliang Deng, Haibo Wang, Sunagawa Masatara, Yan Zhou, Yanqing Liu

**Affiliations:** 1 Institute of Traslational Medicine, Medical College, Yangzhou University, Yangzhou 225001, PR China; 2 Department of Oncology, Yixing Hospital Affiliated to Medical College of Yangzhou University, Yixing, Jiangsu, 214200, PR China; 3 The Key Laboratory of Syndrome Differentiation and Treatment of Gastric Cancer of the State Administration of Traditional Chinese Medicine, Yangzhou, 225001, PR China; 4 Department of Physiology, School of Medicine, Showa University, Tokyo 142, Japan

**Keywords:** CRC, CHIP, Gal1, prognosis, ubiquitination, metastasis

## Abstract

CHIP and Galectin-1 are associated with the development of metastasis in cancer. However, the precise roles of CHIP or Gal1 in colorectal cancer are uncertain. Here, our study explored the relationship and clinical significance of CHIP or Gal1 in CRC. CHIP or Gal1 expression was significantly decreased or up-regulated in CRC compared with adjacent noncancerous tissues by immunohistochemistry on a CRC tissue microarray, respectively. Low CHIP or high Gal1 expression significantly correlated with clinicopathological characteristics in patients, as well as with shorter overall survival. Multivariate Cox regression analysis revealed that CHIP or Gal1 expression was an independent prognostic factor for CRC patients. Moreover, CHIP associated with Gal1 has a synergistic effect on the prediction of CRC prognosis. *In vitro* and *vivo*, high CHIP or low Gal1 expression inhibit CRC growth or metastasis. Our results found that CHIP could degradate Gal1 by ubiquitination. In summary, CHIP could inhibit CRC growth or metastasis through promoting Gal1 ubiquitination and degradation by proteasome. CHIP and Gal1 expressions are novel candidate prognostic markers in CRC. A combined effect of CHIP and Gal1 as efficient prognostic indicators was found for the first time.

## Introduction

Colorectal cancer (CRC) is one of the digestive malignant tumors 1. The incidence of CRC is very different in the world, which ranks first in countries such as North America and Northern Europe. However, the incidence rate is very low in Asia, Africa 2. With the ongoing societal and economic development, the incidence of CRC is becoming more and higher, especially in Asia countries with low incidence. In recent years, the incidence rate is close to the level of European and American countries 3,4.

At present, the treatment of CRC is still based on surgery, chemotherapy, radiotherapy, and molecular targeted drugs 5. Although various clinical treatment methods have made significant progress, the mortality rate of CRC is still gradually increasing, mainly due to local recurrence or distant metastasis 6,7. The development and progression of CRC is an extremely complex process, which contain many oncogenes and tumor suppressor genes 8. If we could find molecular markers in this process to predict the therapeutic efficacy and prognosis of CRC, We are able to prescribe more effective treatments for CRC to reduce probability of recurrence.

CHIP consists of characteristic domains such as TPR, U-box and coiled coil domain 9. CHIP has been known as an E3 ubiquitin ligase, which could degradate many oncoproteins and tumor suppressor protein by ubiquitination in cancers 10. There are more and more studies on the role of ubiquitin-proteasome pathway in tumors 11-13. Every link of ubiquitination maybe become tumor target, which could offer a better method to prdict cancer prognosis or treat cancer.

Gal-1 is a cell-autocrined protein, which plays an important role in the development and metastasis of many tumors 14. Some studies have confirmed that Gal-1 is specifically expressed in many tumor tissues and regulates various biological activities of cancer cells, such as breast cancer, gastric cancer 15,16. However, Gal1 has little research on the role of CRC.

In this article, we focuse on the role of CHIP, Gal1 in CRC and explain that CHIP degrades Gal1 through ubiquitination. Simultaneously, we have proved that CHIP or Gal1 could predict the prognosis of CRC separately. More intriguingly, the combined prediction of the two has a synergistic effect as a novel predictor with more accuracy in survival evaluation.

## Results

### CHIP and Gal1 expression in CRC versus non-cancer tissues

**Figure 1:**
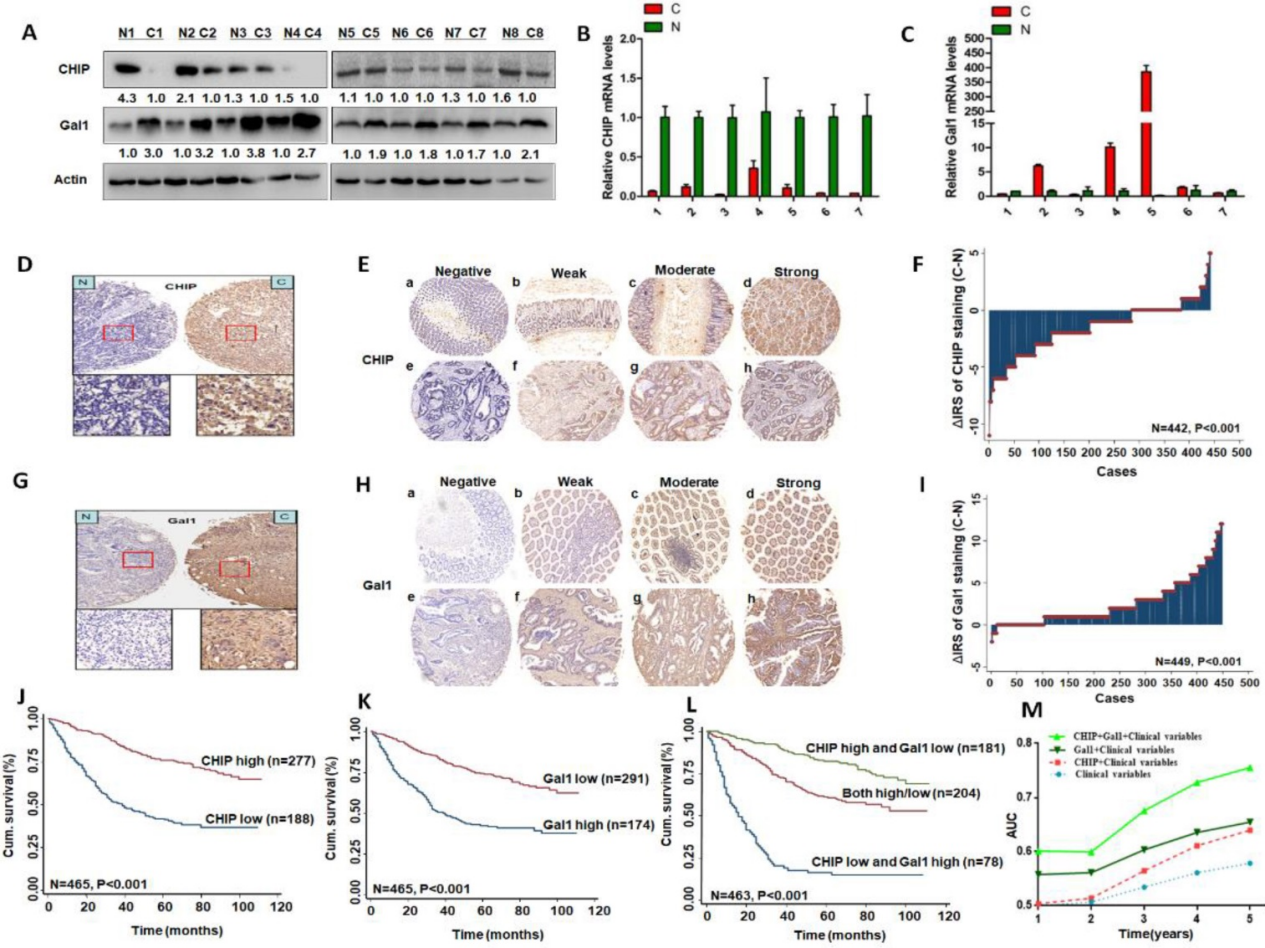
** CHIP or Gal1 expression predicts prognosis of CRC**. **A**: Expression of CHIP and Gal1 protein was detected by Western blot in cancer (C) and normal tissues (N). **B, C**: CHIP and Gal1 mRNA were detected by RT-PCR in cancer (C) and normal tissues (N). **D, G**: CHIP or Gal1 staining in CRC compared with paired normal tissues, respectively. Top panel, original magnification, 40×; bottom panel, magnification, 200×. **E, H:** Representative images of CHIP or Gal1 immunohistochemical staining in TMA. Note: (a-d) Adjacent normal tissue; (e-h) Cancer tissue(a, e, Negative staining. b, f, Weak staining. c, g, Moderate staining. d, h, Strong staining). All panels, original magnification, 40×.** F, I:** The distribution of CHIP or Gal1 staining in TMA compared with paired normal tissues, respectively.** J, K, L:** Kaplan-Meier curves of CHIP, Gal1, and combined with CHIP/Gal1 expression in training cohort for OS. **M:** Time-dependent ROC analyses for clinical risk score (TNM stage, histologic type and tumor diameter), or in combination with CHIP, Gal1, CHIP plus Gal1, respectively. AUC = area under the curve.

We used eight pairs of CRC primary cancer tissues and matched normal tissues to detect protein levels of CHIP and Gal1 by western blotting. Results indicated that CHIP expression were lower in tumor tissues compared with the paired normal tissues, however Gal1 expression were increased (Fig. 1A). CHIP and Gal1 mRNA were detected by RT-PCR. We found that CHIP mRNA were lower and Gal1 mRNA were higher in tumor tissues, compared with corresponding normal tissues respectively (Fig. 1B,C).

Immunohistochemical staining was used to confirm CHIP or Gal1 expression in CRC TMA slides. Representative images of CHIP or Gal1 were showed in TMA in Fig. 1D, 1E and Fig. 1G, 1H, respectively. In TMA of CRC patients, these results showed that CHIP expression was significantly decreased in cancer tissues compared with matched normal tissues(p<0.001; figure 1F). Similarly, we found that Gal1 expression was upregulated in tumor tissues compared with the paired adjacent non-tumor tissues (P < 0.001; Fig. 1I).

### CHIP or Gal1 expression correlates with clinicopathological characteristics

In the CRC cohort, CHIP expression in cancer tissues was significantly correlated with clinicopathological features in Table 1, such as pathological classification, lymph node metastasis, TNM stage, distant metastasis (*p* < 0.05 for all).

As showed in Table 1, we also found that Gal-1 expression in cancer tissues was significantly associated with pathological classification, depth of invasion, lymph node metastasis, TNM stage and distant metastasis (p<0.05 for all).

### Low CHIP or high Gal1 expression correlates with a shorter survival in CRC patients

Kaplan-Meier analysis revealed that low CHIP or high Gal-1 expression in cancer tissues was significantly correlated with a worse 5 year survival of all CRC patients (P < 0.001 and P < 0.001, respectively, log-rank test; Fig. 1J, 1K). Next, univariate and multivariate Cox regression analysis indicated that CHIP or Gal-1 was an independent prognostic factor of CRC patients. Results showed that age, pathological classification, depth of invasion, lymph node metastasis, distant metastasis, TNM stage, CHIP and Gal1 expression were associated with OS of CRC patients by univariate Cox regression analysis (Table 2). In table 3, multivariate Cox regression analysis revealed that CHIP or Gal-1 expression was an independent prognostic factor for CRC patients(CHIP: HR, 0.711, 95% CI, 0.532-0.950, *P* < 0.05; Gal-1: HR, 0.693, 95% CI 0.494-0.972, *P* < 0.05).

### Synergisic effect of CHIP with Gal1 expression on OS in CRC patients

In Fig. 1L, we found that high CHIP expression and low Gal1 expression had a more favorable outcome of survival when compared with CHIP low and Gal1 high expression group or both high/low expression group (*P* < 0.001; log-rank test). Furthermore, high CHIP and low Gal1 expression was alone an effective independent prognostic factor by multivariate Cox regression analysis (*P* < 0.05 for all; Table 3).

To further verify whether CHIP combined with Gal1 had a synergetic effect on the prognosis of CRC patients, we applied clinical risk scores (TNM stage, histologic type and tumor diameter), CHIP expression, Gal1 expression and CHIP plus Gal1 expression to conduct a time-dependent ROC analysis. Our data indicated that the clinical risk scores with CHIP plus Gal1 expression contributed much more than any one of these markers alone in CRC patients (Fig. 1M). For instance, the AUC at year 5 was 0.663 (95% CI, 0.476-0.703) for only clinical risk scores, 0.774 (95% CI, 0.522-0.784) for clinical risk scores combined with CHIP, 0.730 (95% CI, 0.535-0.801) for clinical risk scores combined with Gal1, whereas it was increased to 0.820 (95% CI, 0.607-941) when combined with the clinical risk score and with CHIP plus Gal1 risk score.

### CHIP regulates Gal1 only on protein level

**Figure 2:**
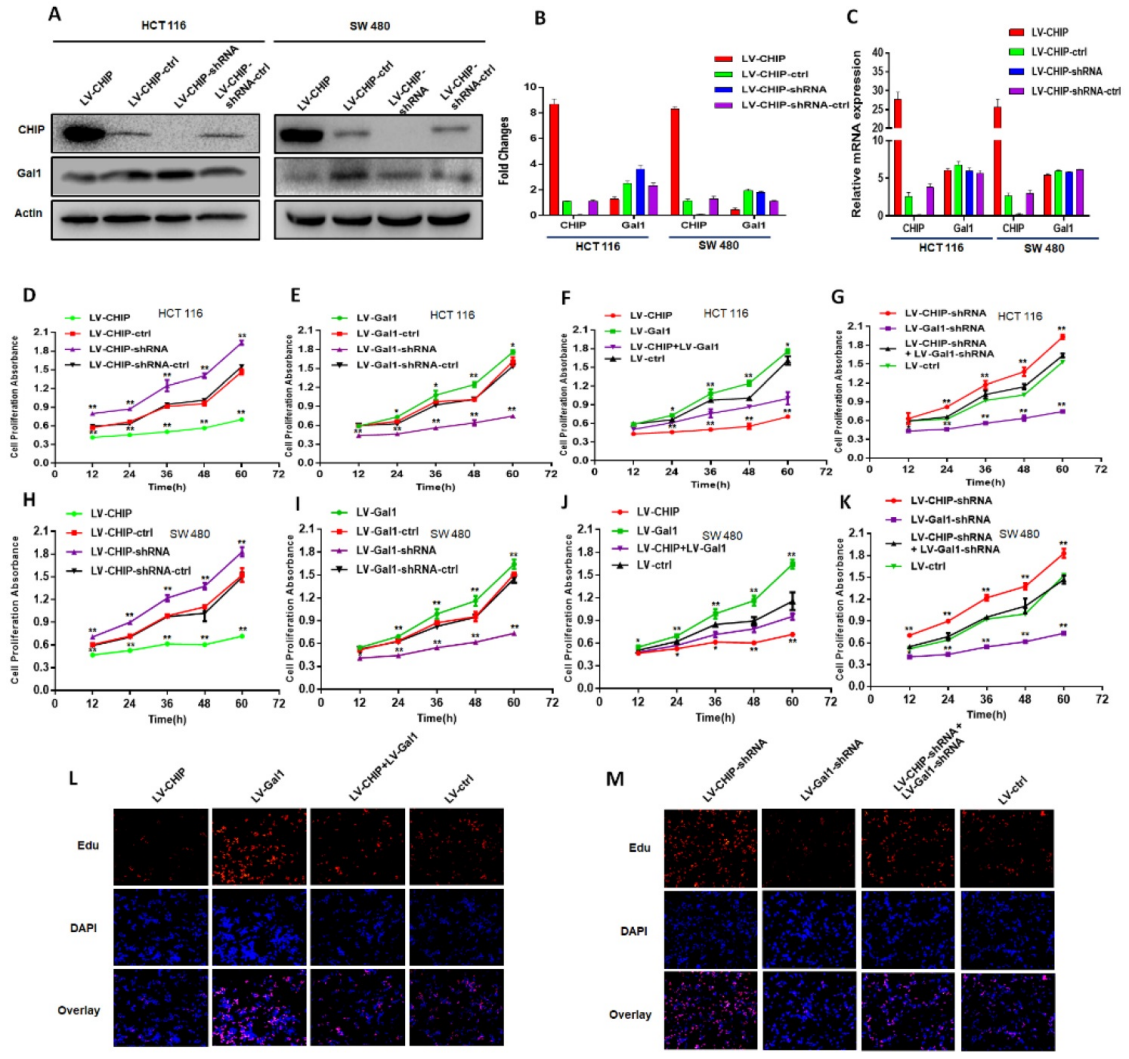
** CHIP suppresses CRC cell growth by decreasing Gal1* in vitro*. A, B**: CHIP protein could negatively regulate Gal1 protein by Western blot. **C:** CHIP mRNA could not regulate MMP-9 mRNA by RT-PCR. **D, E, F, G:** CHIP could inhibit HCT 116 cells proliferation through regulating Gal1 by CCK8 assay(* P < 0.05, ** P < 0.01). **H, I, J, K:** CHIP could inhibit SW 480 cells proliferation through regulating Gal1 by CCK8 assay(* P < 0.05, ** P < 0.01). **L, M:** EdU immunofluorescence assay also proved that HCT 116 cells proliferation capability could be increased by re-infected LV-Gal1-lentivirus in LV-CHIP CRC cell.

We had constructed lentivirus to vary CHIP expression, such as LV-CHIP cells, LV-CHIP-shRNA cells. The lentivirus-mediated overexpression or knockdown of CHIP was analyzed by western blot and RT-PCR. As showed in Fig. 2A, 2B, we found that CHIP could regulate negatively Gal1 on protein level. However, our data indicated that CHIP could not regulate Gal1 on mRNA level by RT-PCR (Fig. 2C).

### CHIP suppresses CRC cell growth by decreasing Gal1

Our CRC data indicated that CHIP or Gal1 was associated with TNM stage. However, we don't know if CHIP or Gal1 could inhibit cell proliferation. Was there a link between them in cell proliferation? For this purpose, Lentiviral was used to generate HCT 116, SW 480 stable cell lines. There were overexpressed LV-CHIP, overexpressed LV-Gal1, lowexpressed LV-CHIP-shRNA, lowexpressed LV-Gal1-shRNA and corresponding controls under normal culture conditions. We performed an CCK8 assay to detect the effect of CHIP and Gal1 on CRC cell growth *in vitro*. Our data indicated that the ability of cell proliferation was significantly increased in LV-CHIP, LV-Gal1-shRNA cell, compared with respective controls. Under the same situation, the trend is reversed in LV-CHIP-shRNA, LV-Gal1 cell (Fig. 2D, 2E; Fig. 2H, 2I; * P < 0.05, ** P < 0.01). Interestingly, we try to make a secondary lentivirus-infection to change Gal1 expression. Results found that the capability of cell proliferation could be increased after we re-infected LV-Gal1-lentivirus to increase Gal1 expression in LV-CHIP CRC cell (Fig. 2F; Fig. 2J; *P< 0.05, **P< 0.01). Relatively, cell proliferation capability of LV-CHIP-shRNA cells reduced after infection with LV-Gal1-shRNA lentivirus (Fig. 2G; Fig. 2K; * P < 0.05, ** P < 0.01).

Meanwhile, we observed the relations of CHIP and Gal1 on HCT 116 cell proliferation by EdU immunofluorescence assay. As in Fig.2L,2M, cell proliferation capability could be increased by re-infected LV-Gal1-lentivirus in LV-CHIP CRC cell. To LV-CHIP-shRNA cell, cell proliferation capability could be reduced by infected LV-Gal1-shRNA lentivirus. Our data indicated that CHIP maybe inhibit CRC cell proliferation through decreasing Gal1.

### CHIP inhibits migration and invasion abilities of CRC cell via regulating Gal1

**Figure 3:**
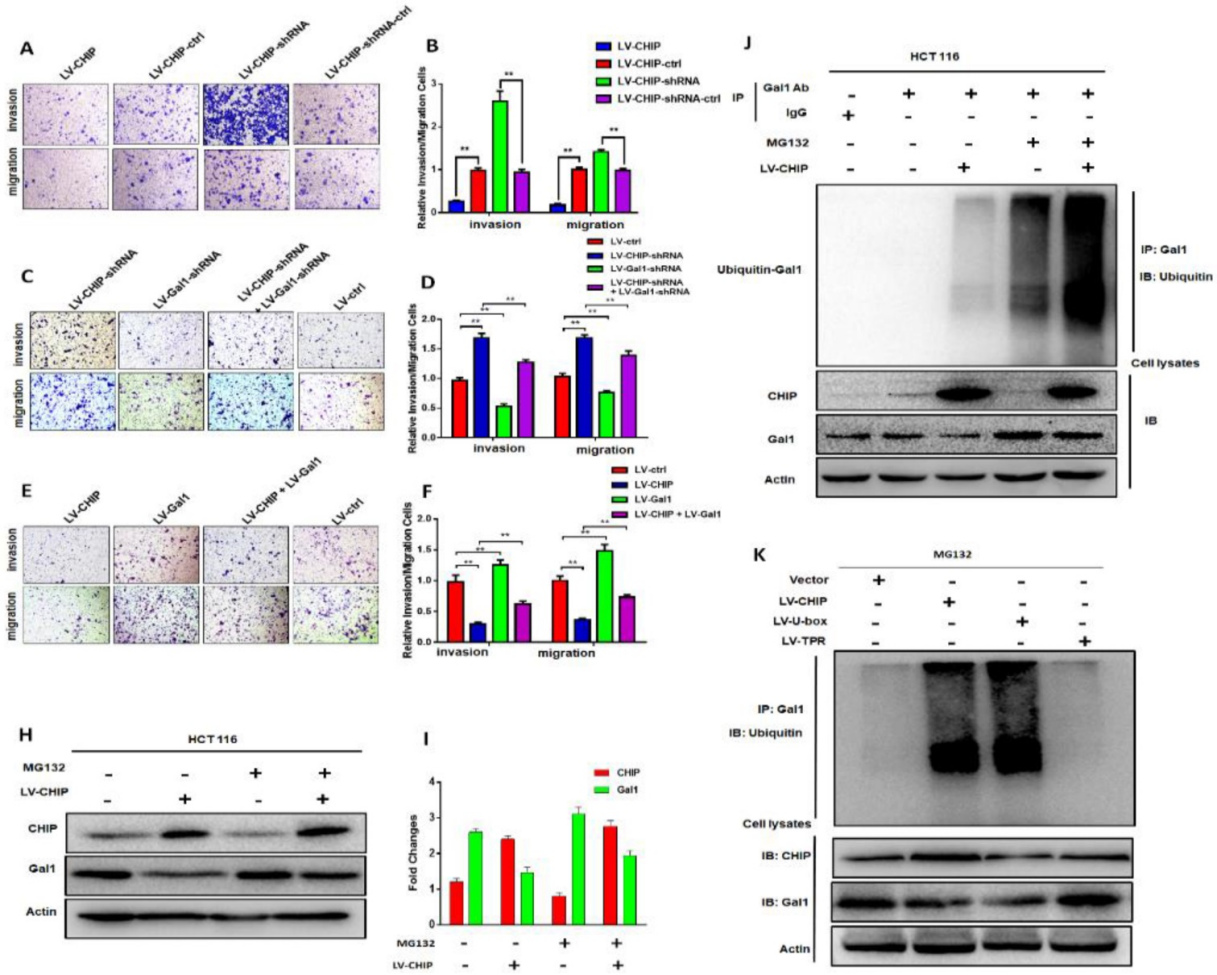
** CHIP inhibits HCT 116 cells migration and invasion via regulating Gal1 *in vitro* and CHIP degradates Gal1 by ubiquitination. A, B:** The migration and invasion ability of HCT 116 cells with different CHIP expression levels was detected by transwell assay. **C, D, E, F:** CHIP could inhibit HCT 116 cells migration and invasion through regulating Gal1 by transwell assay. Note: C, E cresyl violet staining(200× magnification). D, F represent numbers of cells migration and invasion per field (n = 3/group), respectively(* P < 0.05, ** P < 0.01). **H, I:** HCT-116 cells were treated with or without MG132 (10 µM) for 6 h before harvest. Cell lysates were detected by western blot analyses with respective antibodies as shown. **J:** CHIP could degradate Gal1 by ubiquitination: HCT-116 cells were treated with or without MG132(10 µM) for 6 h before harvest. IP with anti-Gal1 antibody or rabbit immunoglobin G as a control group followed by immunoblot with respective antibodies as shown. **k:** U-box domain of CHIP was required for degradation of Gal1: The lentivirus of LV-CHIP, LV-U-box, LV-TPR were transfected into HCT 116 cells. Results indicated that LV-U-box domain could reduce Gal1 levels by ubiquitination.

Previous database analysis had confirmed that CHIP or Gal1 expression was associated with lymph node metastasis, TNM stage and distant metastasis. To further examine the function of CHIP or Gal1 on migration and invasion abilities, we applied transwell assays with HCT 116 cells *in vitro*. Transwell invasion assays showed that the invasion and migration abilities were significantly weakened in LV-CHIP cells. However, LV-CHIP-shRNA cells were significantly enhanced, compared with corresponding control group, respectively (Fig. 3A, 3B, ** *P* < 0.01).

Then, LV-CHIP cells were re-infected lentivirus to vary Gal1 expression. We found that the capability of cell invasion and migration abilities could be improved by re-infected LV-Gal1-lentivirus in LV-CHIP CRC cells (Fig. 3C, 3D; ** *P* < 0.01). Relatively, cell invasion and migration capability of LV-CHIP-shRNA cells reduced after infection with LV-Gal1-shRNA lentivirus (Fig. 3E, 3F; ** *P* < 0.01).

### CHIP promotes Gal1 ubiquitination for degradation via its U-box domain

We had verified that CHIP could negatively regulate Gal1 only on the protein level. To further investigate why CHIP could degrade GAL1 in CRC cells? LV-CHIP CRC cells and corresponding control cells were treated with MG132. Then, western blot showed that the level of Gal1 was restored compared with control group (Fig. 3H, 3I).

As we all know, CHIP is an E3 ligase that could degradate many oncoproteins and suppressor proteins by ubiquitination. To examine whether CHIP mediated ubiquitination of Gal1, we performed an IP-Western experiment to pull down all proteins associated with GAL1 and make an ubiquitination assay. We found that CHIP significantly promoted ubiquitination of Gal1 protein (Fig. 3J). Furthermore, we determined which ubiquitinated function domain of CHIP was required for degradation of Gal1. The lentivirus of LV-CHIP, LV-U-box, LV-TPR were transfected into HCT 116 cells. Results indicated that LV-U-box cells could reduce Gal1 levels by ubiquitination as same as LV-CHIP cells, but LV-TPR cells had no effect (figure 3K). All results showed that CHIP functioned as an E3 ligase to degradate ubiquitination of Gal1 by its U-box domain.

### CHIP suppresses CRC cell growth and metastasis *in vivo*

**Figure 4:**
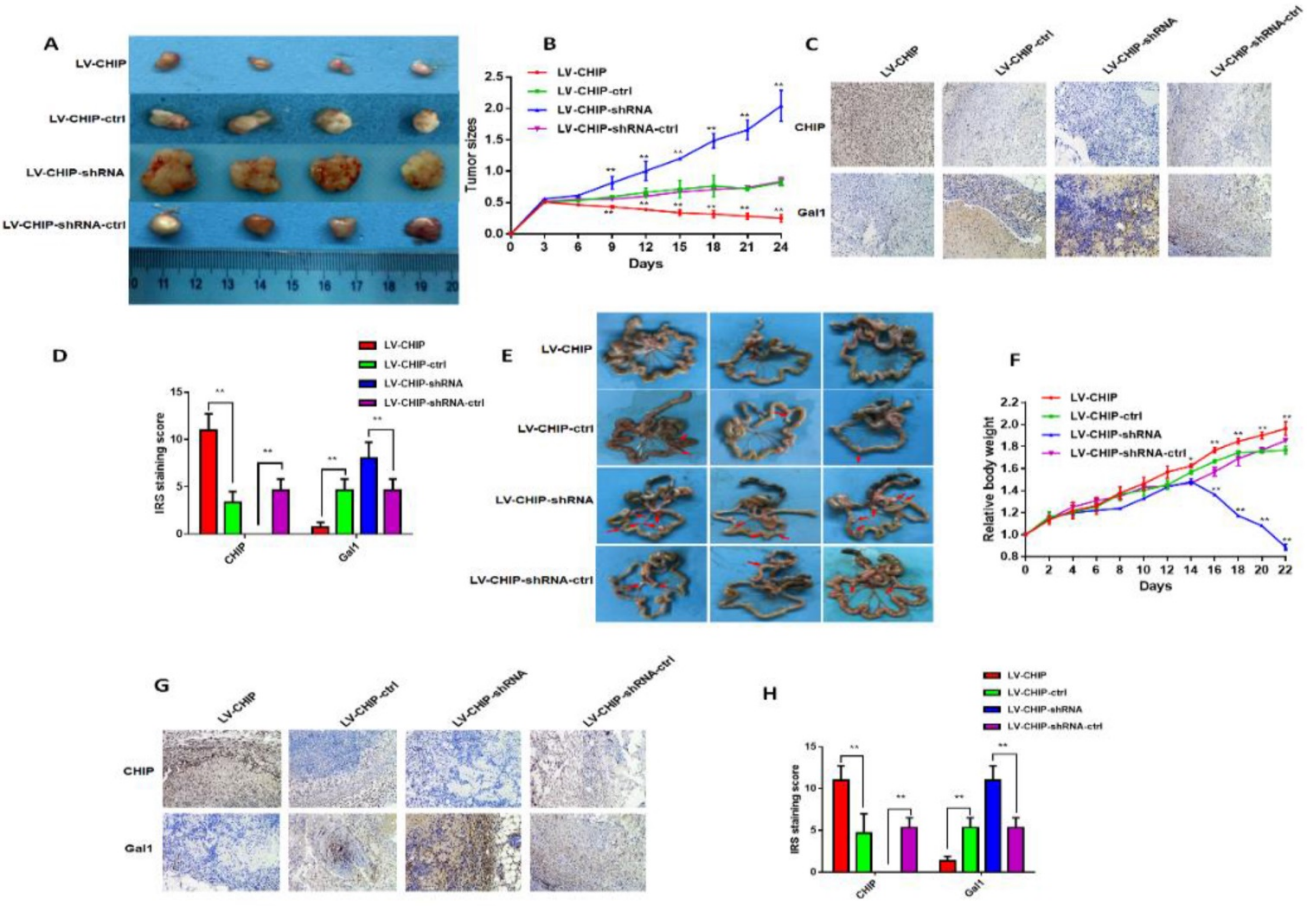
** CHIP suppresses CRC cell growth and metastasis *in vivo*. A:** Representative photographs of the tumor size were captured four groups. Tumor size was significantly smaller in LV-CHIP group, larger in LV-CHIP-shRNA group compared with the control group, respectively. **B:** The tumor size was calculated in four groups every three days. The tumor size was significantly larger in LV-CHIP group, smaller in LV-CHIP-shRNA group compared with the control group, respectively (**P < 0.01). **C, D:** The expressions of CHIP, Gal1 in tumor tissues were tested by IHC. The IRS staining scores of CHIP, Gal1 were evaluated (n = 3). Data were presented as mean ± SD (**P < 0.01). **E:** Representative photographs of metastatic nodules of the peritoneal cavity in four groups. Over-expressed CHIP could inhibit metastasis of CRC cells. In LV-CHIP-shRNA group, metastasis of CRC cells reduced much more than in control group. **F:** The weights of mice in four groups were monitored every 2 days. After two weeks, the mice weights higher in LV-CHIP group, lower in LV-CHIP-shRNA group compared with the control group, respectively(**P < 0.01). **G, H:** The expressions of CHIP, Gal1 in metastatic nodules of the peritoneal cavities were tested by IHC. The IRS staining scores of CHIP, Gal1 were evaluated (n = 3). Data were presented as mean ± SD (**P < 0.01).

To further investigate the function of CHIP on inhibiting cell proliferation *in vivo*, stable LV-CHIP, LV-CHIP-ctrl, LV-CHIP-shRNA and LV-CHIP-shRNA-ctrl HCT 116 cells were injected subcutaneously into nude mice. The data showed that tumour growth was suppressed in the LV-CHIP group, however increased in the LV-CHIP-shRNA, compared with corresponding control group (figure 4A). Tumor growth was monitored every three days. The tumor size was significantly larger in LV-CHIP group, smaller in LV-CHIP-shRNA group compared with the control group, respectively (Fig. 4B; **P < 0.01).Simultaneously, we detected the protein expressions of CHIP, Gal1 in the xenograft tumors by IHC. The results showed that CHIP expression in tumors were higher or lower in LV-CHIP group or LV-CHIP-shRNA group than the respective controls. CHIP expression was negatively associated with Gal1 expression (Fig. 4C, 4D; **P < 0.01).

In addition, we also used these stable CHIP expressed HCT 116 cells to inoculate into the peritoneal cavity of BALB/c nude mice. We found that abdominal tumor metastasis was more or less in LV-CHIP group or LV-CHIP-shRNA group, compared with the corresponding control group respectively (Fig. 4E). The weight of per mouse was monitored every 2 days. We found that the relative weight of LV-CHIP-shRNA group reduced significantly at days 16, 18, 20 and 22 compared with the control group, whereas the weight of LV-CHIP group increased at days 14, 16, 18, 20 and 22 compared with the control group(Fig. 4F; *P < 0.05, **P < 0.01). CHIPS, Gal1 expression in metastatic tumor were also detected by IHC. Our results indicated that CHIP expression positively associated with Gal1 expression (Fig. 4G, 4H; **P < 0.01). All results revealed that CHIP could inhibit CRC cell growth and metastasis via regulating Gal1 *in vivo*, which was the same result as *in vitro*.

## Discussion

CRC is a common clinical gastrointestinal malignant tumor. In recent years, epidemiological studies have shown that the incidence of colorectal cancer has younger trend 17. With the development of various medical technologies, the clinical treatment effect has been greatly improved on CRC patients. However, local recurrence or distant metastasis is easy to appear after surgery, the survival rate of most patients has not been improved from the perspective of long-term survival rate 18. CRC is a multi-gene, multi-stage, long-term complex pathological process. Therefore, finding new effective markers to predict the prognosis of CRC is important to improve the survival rate in the complex molecular regulation network.

Studies have confirmed that ubiquitination is an important posttranslational modification 19. In clinical researches, ubiquitinated dysfunction could upregulate many oncogenic proteins that result in tumor formation 20-22. CHIP is a typical representative of ubiquitin ligase E3, which has suppressed tumor by inducing ubiquitination to degradate many oncogenic proteins. Few studies had explored CHIP expression related to progression and prognosis of gastric cancer 23, breast cancer 24. In the present study, we found that CHIP expression in CRC tissues was lower compared with matched adjacent normal tissues. CHIP could be an effective marker for lymph node metastasis, TNM stage, distant metastasis and OS in CRC patients. Using cell and animal model experiments, we confirmed that CHIP could inhibit CRC cells proliferation, migration and invasion *in vivo* or *vitro*. Moreover, CHIP was an independent positive prognostic factor for CRC patients by univariate and multivariate Cox proportional hazards regression analysis.

Gal1 is an important member of the galectin family. It could regulate apoptosis and cell differentiation, bind with CD45, CD3 and CD4 to inhibit CD45 protein phosphatase activity and lymphocyte-activated enzyme dephosphorylation 25. Studies confirmed that Gal-1 was closely related with tumor development, metastasis, invasion and malignancy, such as gastric cancer 26, ovarian cancer 27, and pancreatic cancer 28. In this study, our data indicated that Gal1 could promote CRC cells proliferation, migration and invasion. Simultaneously, we found that Gal1 was a negative marker of predictive prognosis for CRC patients via database analysis.

CHIP is one of the E3 ubiquitin ligases, which possessed ubiquitin ligase activity to degradate many more oncoproteins. In our study, we had proved that CHIP inhibited CRC cells proliferation and metastasis through ubiquitinated targeted-regulating Gal whether *in vivo* or* vitro*. From our CRC database analysis, we had drawn a conclusion that CHIP or Gal1 was independent predictive prognostic marker, respectively. Next, we tried to analyze whether these two indicators had joint synergy in predicting prognosis of CRC. Excitingly, CHIP and Gal1 had a synergistic effect by Kaplan-Meier survival analysis and clinical factor component ROC curve analysis. Using cox regression model had confirmed that high CHIP and low Gal1 expression was a most effective independent prognostic factor in all groups.

In summary, we have demonstrated that CHIP or Gal1 is prognostic molecular biomarker for CRC patients. CHIP could suppress CRC cells cells proliferation and metastasis through ubiquitinated targeted-regulating Gal *in vivo* and *in vitro*. Most noticeably, we first revealed that the combined value of CHIP and Gal1 as efficient prognostic factors had synergistic effect. To further study these two proteins role maybe provide new opportunities for exploitation in novel therapeutic colorectal strategies.

## Materials and Methods

### Patient specimens and tissue samples

The CRC database of 470 patients contained detailed pathological data and survival follow-up time. Tissue samples and patient specimens were collected in Yixing Hospital affiliated to Yangzhou University Medical College. These patients were admitted to the department of oncology from 2006.01 to 2010.12, and were followed up at least 5 years. The clinicopathological features were described in Table S1. Overall survival was the primary endpoint and the survival time was calculated from the date of surgery to the date of death or to the final follow-up.

The Ethics Committee of Yixing Hospital approved all subjects in this study, which was performed according to the principles of the Declaration of Helsinki.

### Construction of Tissue Microarray (TMA) and Immunohistochemistry

The tumor tissue paraffin blocks were selected and the tissues were first verified by HE staining. Then, the cancer tissues and corresponding adjacent tissues were used for TMA construction. In brief, each spot had a 1.5mm diameter to contain tumor block and corresponding nontumoral tissues. TMA chip blocks were placed in a 55°C incubator for 10 minutes and cooled at room temperature. These chip blocks were carried out in a cryostat at 4 μm thickness. The slices were transferred to extend at 45 ℃ water for 2 min, baked 58 ℃ for 18 h, and stored at -20 °C for later use.

The immunostaining protocol was described earlier 29. Rabbit monoclonal antibodies anti-CHIP (1:100, Cell. Signaling Technology, MA USA) and Gal1 (1:100, Epitomics, California, USA) were incubated at 4℃ overnight. The staining scores of the tissue controls were pre-evaluated as a quality control of immunostaining in each microarray slide.

### Evaluation of immunostaining

The staining of CHIP or Gal1 in the tissue was scored by two pathologists blinded to the clinical data. The training cohort was evaluated by the semi-quantitative immunoreactivity score (IRS), as reported elsewhere 30. Receiver operator characteristic (ROC) analysis was used to determine the optimum cutoff value of CHIP or Gal-1 IRS according the area under the curve (AUC) for 1, 3 and 5 years of OS. The optimum cutoff points of CHIP IRS were showed to be 4 since it had the best predictive value for survival (Fig. S1A). In this case, CHIP expression is defined as low or high expression in tumor tissues with IRS 0-3 or IRS 4-12, respectively. Similarly, the optimum cutoff points of Gal1 IRS were showed to be 6 (Fig. S1B), tissues with IRS 0-5 or 6-12 were classified as low or high expression of Gal1, respectively.

### Cell lines and animals

HCT 116 and SW 480 cells were obtained from Wuhan procell life Science and Technology Co, Ltd. The cells RPMI-1640 medium supplemented with 10 % FBS and 1 % penicillin/streptomycin. All the cells were incubated under 37 ℃, 5 % CO_2_ condition.

Female BALB/c nude mice were offered from the Comparative Medicine Laboratory Animal Center [License No. scxk (SU) 2012-0004] of Yangzhou University. The mice (6-8 weeks old) were settled down in specific pathogen-free conditions and cared according with the National Institutes of Health Guide for the Care and Use of Laboratory Animals.

### Lentiviral infection and generation of stable cell lines

HCT 116, SW 480 cells were infected with LV-CHIP, LV-CHIP-ctrl, LV-CHIP-shRNA and LV-CHIP-shRNA-ctrl (LV-Gal1, LV-Gal1-ctrl, LV-Gal1-shRNA and LV-Gal1-shRNA-ctrl) at a MOI of 20 plus 10 μg/ml of Polybrene (GeneChem, Shanghai, China), respectively.

HCT 116, SW 480 cells were maintained with normal RPMI-1640 culture medium for 24 h after lentiviral infection 8 h. Subsequently, the cells were incubated in RPMI-1640 with 2 μg/ml puromycin (Gibco-BRL, Gaithersburgh, MD, USA). Using Western blot assay verified the knockdown and overexpression efficiency of CHIP or Gal1 (Fig. S1C, S1D).

### CCK8 assay

HCT 116 or SW 480 stable cell lines were seeded in 96-well plates at a density of 8000 cells/well. At five time points(12, 24, 48, 60, and 72 hours), Cell Counting Kit-8(CCK-8) solution(Dojindo Molecular Technology Inc, Shanghai, China) was used to detect cells growth. The absorbance at 450 nm was measured by an automatic microplate reader.

### EdU immunofluorescence assay

HCT 116 or SW 480 stable cell lines were cultured to logarithmic growth phase. 5×10^3^ cells/100μl/well were seeded in a 96-well plate. After 24 hours, immunofluorescence analysis based on the protocol of Edu Kit (RIBOBIO CO, LTD, Guang Zhou, China).

### Cell migration and invasion assay

Transwell invasion assay was performed based on a previously reported protocol 31. Briefly, the transwell filter inserts were coated without or with matrigel(Millipore, Billerica, MA, USA) for cell migration and invasion assays, respectively. HCT 116 stable cells were seeded at a concentration of 5×105 cells/100μl onto the upper chamber of Transwell filters (8 μm pore size, Millipore, Billerica, MA, USA). 500μl RPMI1640 medium contained 10% FBS was introduced into the lower chamber. After 24 hours incubation, the invaded cells were fixed in methanol and stained with 0.1% crystal violet. The number of invaded cells was counted under the inverted microscope and photographed in five different fields of each well.

### Western blot and immunoprecipitation

Additive proteins were extracted from CRC tumor tissues or cells. Using the bicinchoninic acid method to detect protein concentrations. 80 μg proteins per hole were separated on 10% SDS-PAGE gels. Then transferred to membranes, incubated antibody and so on. The protocols were performed as previously described 29. The monoclonal rabbit anti-CHIP (1:1000, Cell Signaling Technology California, USA), monoclonal rabbit anti-Gal1(1:1000, Epitomics, MA, USA), and monoclonal mouse anti-β-actin antibody(1:2000; Beyotime Biotechnology, Nantong, China) were used for the primary antibody. the protein bands intensity were quantified by using Image J software (version 1.44, Wayne Rasband, National Institutes of Health, USA), after normalization to the corresponding β-actin level.

For immunoprecipitation, the protocol of Protein A/G PLUS-Agarose Immunoprecipitation Reagent (SANTA CRUZ BIOTECHNOLOGY, INC) was used to seek all proteins that bind to Gal1. Briefly, Gal1 antibodies (2 μg) and 20 μl Protein A/G PLUS-Agarose beads were added to the cell lysates. The mixture was rotated at 4 °C for 24 h. Immunoprecipitates were collected by centrifugation at 2,500 rpm for 5mins at 4 °C. The precipitated complexes and cell lysates were detected for western blot analyses.

### Quantitative Real-Time PCR

Real-Time PCR RNAs were isolated using RNeasy Mini Kit (Invitrogen, Carlsbad, USA) according manufacturer's manual steps. The purified RNAs were reversely translated into cDNA using a RevertAid RT reverse transcription kit (Thermo Fisher Scientific, Waltham, MA, USA). The transcriptional cDNAs were then carried out with SYBR Green I-based real-time quantitative PCR analysis by using an Applied Biosystems 7500 Real-time PCR System (Roche Applied Science, Penzberg, Upper Bavaria, Germany).

The PCR primers were used as follows: (CHIP-F 5'- CGA TCA CCC GGA ACC CGC T -3' and CHIP -R, 5'- CCA GGC TGT AAG CTC GCT GC - 3'; Gal1-F 5'- GCG TGG CTG CTG GGA GGT ATC -3' and Gal1-R 5'- GGA ACA GAA AGA CTC CAA TG - 3'; β-actin-F, 5'- CAA CGA ATT TGG CTA CAG CA -3' and β-actin--R, 5'- AGG GGT CTA CAT GGC AAC TG -3' (Sangon Biotechnology Inc., Shanghai, China). CHIP or Gal1 mRNA was normalized to an internal control β-actin and analyzed by using the ΔΔCt method. All reactions were performed in duplicate.

### Tumor xenograft and abdominal metastasis model

In tumor xenograft model, HCT 116 stable cell lines (0.2ml 1×10^7^ cells/mouse; 5 mice/group) were inoculated subcutaneously in the flanks of BALB/c nude mice. After 21 days, the mice were sacrificed and tumors were excised and photographed. All the tumor tissues were divided into 10 % buffered formalin.

HCT 116 stable cell lines were injected into the peritoneal cavity of BALB/c nude mice in the peritoneal metastasis model. Mice were killed for the peritoneal metastasis after 28 days. Intraperitoneal metastatic tumors were displayed and taken a photograph, fixed in 10 % formalin.

All the animal experiments were performed with the institutional ethical requirements and approved by the Committee of YangZhou University for the Use and Care of Animals.

### Statistical analysis

The correlations between CHIP, Gal1 expression and clinicopathological data were evaluated by Fisher's exact test.The IRS of CHIP, Gal1 expression were assessed in tumors and corresponding non-tumours by Wilcoxon test (grouped). OS had a difference by Kaplan-Meier survival analysis. Univariate or multivariate Cox regression analysis was used to estimate the HRs and 95% CI. We use the STATA software (version 10.1; StataCorp, College Station, TX) to analyze all experimental data. P < 0.05 was considered statistically significant.

## Supplementary Material

Supplementary figure and table.

## Figures and Tables

**Table 1:** Relationship between expression levels of CHIP or Gal1 and clinicopathological features in CRC patients

Variables	CHIP				Gal1		
	n=465 cases				n=465 cases		
	low (%)	high (%)	P^a^		low (%)	high (%)	P^a^
**All patients**	188 (40.4)	277 (59.6)			291 (62.6)	174 (37.4)	
**Age (years)**			**0.117**				**0.137**
≤ 65	100 (37.9)	164 (62.1)			172 (64.9)	93 (35.1)	
>65	88 (46.8)	113 (53.2)			119 (59.5)	81 (40.5)	
**Gender**			**0.08**				**0.315**
Males	105 (37.6)	174 (62.4)			171 (61.5)	107 (38.5)	
Females	83 (44.6)	103 (55.4)			120 (64.2)	67 (35.8)	
**Pathological classification^b^**			**0.039**				**0.007**
I	3 (60.0)	2 (40.0)			3 (60.0)	2 (40.0)	
II	162 (38.7)	257 (61.3)			270 (64.4)	149 (35.6)	
III	21 (58.3)	15(41.7)			14 (38.9)	22(61.1)	
**Depth of invasion^b^**			**0.064**				**<0.001**
T1/T2	49 (47.6)	54 (52.4)			86 (84.3)	16 (15.7)	
T3/T4	138 (38.5)	220 (61.5)			201 (56.0)	158 (44.0)	
**Lymph node metastasis^b^**			**<0.001**				**<0.001**
N0	84 (30.7)	190 (69.3)			200 (73.3)	73 (26.7)	
N1/N2	103 (54.8)	85 (45.2)			88 (46.6)	101 (53.4)	
**TNM stage^b^**			**<0.001**				**<0.001**
I	39 (44.3)	49 (55.7)			73 (83.9)	14 (16.1)	
II	41 (23.2)	136 (76.8)			124 (70.1)	53 (29.9)	
III	94 (52.8)	84 (47.2)			88 (49.2)	91 (50.8)	
IV	13 (76.5)	4 (23.5)			2 (11.8)	15 (88.2)	
**Tumor diameter^b^**			**0.429**				**0.052**
≤ 5 cm	152 (40.6)	222 (59.4)			241 (64.4)	133 (35.6)	
>5 cm	35 (38.9)	55 (61.1)			49 (54.4)	41 (45.6)	
**Distant metastasis**			**0.003**				**<0.001**
M0	174(39.0)	272(61.0)			289(64.8)	157(35.2)	
M1	14(73.7)	5(26.3)			2(10.5)	17(89.5)	

a Two-sided Fisher's exact tests b Some patients missing these clinical pathological parameters

**Table 2:** Univariate Cox regression analysis of CHIP or Gal1 expression and clinicopathological variables predicting survival in patients with CRC patients

Variables	n=470 cases	
	HR (95 % CI)	P
Age (≤65 vs. > 65)	1.607 (1.215-2.126)	**0.001**
Gender (male vs. female)	1.013 (0.762-1.347)	**0.927**
Pathological classification (I/II vs. III)	2.475 (1.587-3.860)	**<0.001**
Depth of invasion (T1/T2 vs. T3/T4)	3.687 (2.270-5.990)	**<0.001**
Lymph node metastasis (N0 vs. N1/N2)	2.807 (2.112-3.731)	**<0.001**
TNM stage (I/II vs. III/IV)	3.214 (2.407-4.291)	**<0.001**
Distant metastasis(M0 vs. M1)	8.150 (4.849-13.699)	**<0.001**
Tumor diameter (≤5 cm vs. >5 cm)	1.196 (0.848-1.688)	**0.307**
CHIP expression (low vs. high)	0.314 (0.235-0.419)	**<0.001**
Gal1 expression (low vs. high)	0.373 (0.281-0.495)	**<0.001**

**Table 3:** Multivariate Cox regression analysis of CHIP, Gal1, CHIP/Gal1 expression and clinicopathological variables predicting survival in patients with CRC

Variables	HR (95% CI)	P^a^
**CHIP**		
Gender (male vs. female)	0.939 (0.702-1.256)	0.672
Pathological classification (I/II vs. III)	1.882 (1.175-3.015)	0.009
TNM stage (I/II vs. III/IV)	3.147 (2.335-4.241)	<0.001
Tumor diameter (≤5 cm vs. >5 cm)	1.112 (0.771-1.603)	0.571
CHIP expression (low vs. high)	0.711 (0.532-0.950)	**0.021**
**Gal1**		
Gender (male vs. female)	0.941 (0.703-1.259)	0.683
Pathological classification (I/II vs. III)	1.885 (1.178-3.016)	0.008
TNM stage (I/II vs. III/IV)	3.091 (2.293-4.168)	<0.001
Tumor diameter (≤5 cm vs. >5 cm)	1.108 (0.769-1.597)	0.582
GAL1 expression (low vs. high)	0.693 (0.494-0.972)	**0.034**
**CHIP/ Gal1**		
Gender (male vs. female)	0.936 (0.699-1.252)	0.655
Pathological classification (I/II vs. III)	1.860 (1.163-2.974)	0.010
TNM stage (I/II vs. III/IV)	3.140 (2.329-4.234)	<0.001
Tumor diameter (≤5 cm vs. >5 cm)	1.135 (0.788-1.635)	0.135
**CHIP/ Gal1 expression**		
CHIP high and Gal1 low vs. Both low/high	1.762(1.012-3.067)	**0.045**
CHIP high and Gal1 low vs CHIP low and Gal1 high	0.633(0.442-0.908)	**0.013**

^a^Multivariate Cox regression analysis including gender, pathological classification, TNM stage, tumor diameter, CHIP or Gal1 or combined 2 proteins expression status.

## Abbreviations

CRC: colorectal cancer
Gal1: Galectin-1
OS: overall survival
TMA: Tissue Microarray
AUC: area under the curve
CCK-8: Cell Counting Kit-8
