# Supplementary material for: CHIP-mediated ubiquitination of Galectin-1 predicts colorectal cancer prognosis
Source: Int J Biol Sci. 2020 Jan 14;16(4):719–29. doi: 10.7150/ijbs.41125 (PMC6990922; doi:10.7150/ijbs.41125)
Supplement: Supplementary file 1 — Supplementary figure and table. [file ijbsv16p0719s1.pdf]

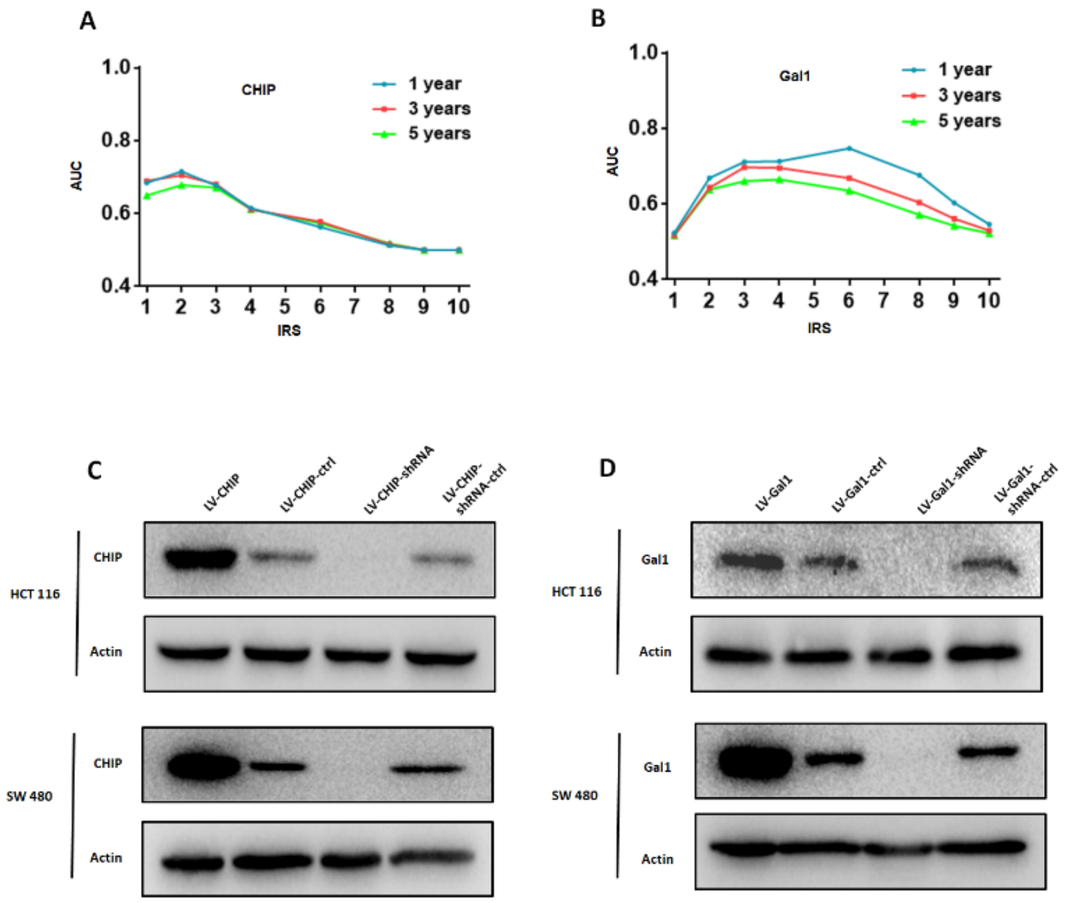


Fig. S1 **A, B:** The cut-off values of CHIP or Gal1 for immunoreactivity score(IRS) were calculated for 1, 3 and 5 years of OS time according the area under the curve(AUC) . **C, D:** HCT 116, SW 480 cells were infected with LV-CHIP, LV-CHIP-ctrl, LV-CHIP-shRNA and LV-CHIP-shRNA-ctrl(LV-Gal1, LV- Gal1-ctrl, LV-Gal1-shRNA and LV-Gal1-shRNA-ctrl) at a MOI of 20 plus 10 μg/ml of Polybrene, respectively. Using Western blot assay verified the knockdown and overexpression efficiency of CHIP or Gal1.

Table S1 the patients' clinicopathologic information in CRC

| Variables | | n | |
|---|---|---|---|
| All patients | | 470 | (%) |
| Age (years) | | | |
| | ≤65 | 267 | 56.8 |
| | ＞65 | 203 | 43.2 |
| Gender | | | |
| | Males | 281 | 59.8 |
| | Females | 189 | 40.2 |
| Pathological classification[b] | | | |
| | Ⅰ | 5 | 1.1 |
| | Ⅱ | 423 | 91.2 |
| | Ⅲ | 36 | 7.7 |
| Depth of invasion[b] | | | |
| | T1 | 9 | 2.0 |
| | T2 | 94 | 20.2 |
| | T3 | 347 | 74.6 |
| | T4 | 15 | 3.2 |
| Lymph node metastasis[b] | | | |
| | N0 | 276 | 59.2 |
| | N1 | 126 | 27.0 |
| | N2 | 64 | 13.8 |
| TNM stage[b] | | | |
| | Ⅰ | 88 | 18.9 |
| | Ⅱ | 179 | 38.6 |
| | Ⅲ | 180 | 38.8 |
| | Ⅳ | 17 | 3.7 |
| Tumor diameter[b] | | | |
| | ≤ 5 cm | 378 | 80.6 |
| | ＞5 cm | 91 | 19.4 |
| Distant metastasis | | | |
| | M0 | 451 | 95.9 |
| | M1 | 19 | 4.1 |

b Some patients missing these clinical pathological parameters
